# Infections of virulent and avirulent viruses differentially influenced the expression of *dicer-1, ago-1*, and microRNAs in *Bombus terrestris*

**DOI:** 10.1038/srep45620

**Published:** 2017-04-04

**Authors:** Jinzhi Niu, Ivan Meeus, Dieter IM De Coninck, Dieter Deforce, Kayvan Etebari, Sassan Asgari, Guy Smagghe

**Affiliations:** 1 Key Laboratory of Entomology and Pest Control Engineering, College of Plant Protection, Southwest University, Chongqing 400716, China; 2 Department of Crop Protection, Faculty of Bioscience Engineering, Ghent University, Coupure Links 653, B-9000 Ghent, Belgium; 3 Laboratory for Pharmaceutical Biotechnology, Faculty of Pharmaceutical Sciences, Ghent University, Ottergemsesteenweg 460, B-9000 Ghent, Belgium; 4 Australian Infectious Disease Research Centre, School of Biological Sciences, The University of Queensland, Brisbane, QLD 4072, Australia

## Abstract

The microRNA (miRNA) pathway is well established to be involved in host-pathogen interactions. As key insect pollinators, bees are suffering from widely spreading viruses, especially honeybees and bumblebees. In order to better understand bee-virus interaction, we comparatively analyzed the involvement of the bumblebee miRNA pathway upon infection by two different viruses. In our setup, an avirulent infection is induced by slow bee paralysis virus (SBPV) and a virulent infection is induced by Israeli acute paralysis virus (IAPV). Our results showed the increased expressions of *dicer-1* and *ago-1* upon SBPV infection. There were 17 and 12 bumblebee miRNAs differentially expressed upon SBPV and IAPV infections, respectively. These results may indicate the involvement of the host miRNA pathway in bumblebee-virus interaction. However, silencing of *dicer-1* did not influence the genome copy number of SBPV. Target prediction for these differentially expressed miRNAs showed their possible involvement in targeting viral genomic RNA and in the regulation of networks in bumblebee. Our study opens a new insight into bee-virus interaction meditated by host miRNAs.

RNA interference (RNAi) is a conserved mechanism for posttranscriptional gene silencing to regulate gene expression and defense against viral infection in insects. Currently, three sub-pathways are recognized under the RNAi pathway based on the produced small RNAs, including: small interfering RNA (siRNAs), microRNAs (miRNA), and Piwi-interacting RNAs (piRNAs) pathways. In bees, two aspects of the siRNA-pathway-mediated antiviral response has been considered: virus derived siRNAs and utilization of this pathway to control virus through virus-specific dsRNA^1^. Our previous study in the bumblebee *Bombus terrestris* showed that Israeli acute paralysis virus (IAPV) replicated very fast and acted as a very virulent virus whereas slow bee paralysis virus (SBPV) also replicated fast but induced no mortality after injection; furthermore, both IAPV and SBPV infections could induce the expression of *dicer-2*, while IAPV, but not SBPV, infection triggered the production of predominantly 22 nt-long virus-derived siRNAs^2^.

As another important sub-pathway of RNAi in insects, the miRNA pathway has been documented to be involved in different aspects of development, such as formation of germ cells, wing and muscle, neurogenesis, apoptosis, and phenotypic plasticity^3^, while this pathway is also well established to be involved in host-pathogen interactions^3^,^4^. The canonical biogenesis of miRNAs initiates in the nucleus where monocistronic, bicistronic or polycistronic transcripts are produced. These contain stem-loop structures known as the primary miRNA (pri-miRNA). The pri-miRNA is cleaved by Drosha and Pasha to liberate the precursor miRNA (pre-miRNA). After exportation to the cytoplasm, the hairpin head of the pre-miRNA stem-loop is cut by Dicer-1 to yield a miRNA duplex. The miRNA duplex is then loaded into RNA induced silencing complex (RISC), which typically includes Argonaut 1 (Ago-1), resulting in the degradation of one of the strands. Then, the mature miRNA binds to the target mRNA and leads to mRNA degradation or translational repression^5^. Besides, there are also non-canonical pathways of miRNA biogenesis, which are Drosha-independent but can be Dicer-dependent or Dicer-independent^6^. The production and regulatory effects of miRNAs on insect-virus interactions could be complex. The first layer of complexity relates to the origin of miRNAs, which could be derived from the host or the virus. The second layer of complexity arises from the two-way interplay, meaning host-encoded miRNAs can target genes from both the host and the virus, and *vice versa* for virus encoded miRNAs^7^.

Differential expression of miRNAs has been associated with honeybee development and social behaviors^8^,^9^,^10^,^11^. Recently, with the genome sequencing of two bumblebee species, *Bombus terrestris* and *B. impatiens*, two datasets of miRNAs have been annotated for both species^12^. However, the regulatory effect of the miRNA pathway on bee-virus interaction is still unknown. Thus, in the current report, we comparatively analyzed the involvement of bumblebee miRNAs upon infections of an avirulent virus SBPV and a virulent virus IAPV. First, we analyzed the expression of the core genes (*dicer-1* and *ago-1*) of the miRNA pathway upon virus infection. Secondly, through small RNA sequencing, we analyzed the miRNA profiles following viral infections. To have a further insight into miRNA-mRNA interaction, we predicted the possible targets for these miRNAs. Finally, we silenced *dicer-1* to analyze the outcome of SBPV infection. Our study showed different features of the miRNA pathway response between the infection by the avirulent and the virulent virus, which may be associated with the virulence of the bee viral infections. In addition, these initial data could be the starting point to understand the function of miRNAs in cell regulatory systems of bee-virus interactions.

## Results and Discussion

### Significant effects of SBPV and IAPV infections on the expressions of *dicer-1* and *ago-1*

**Figure 1:**
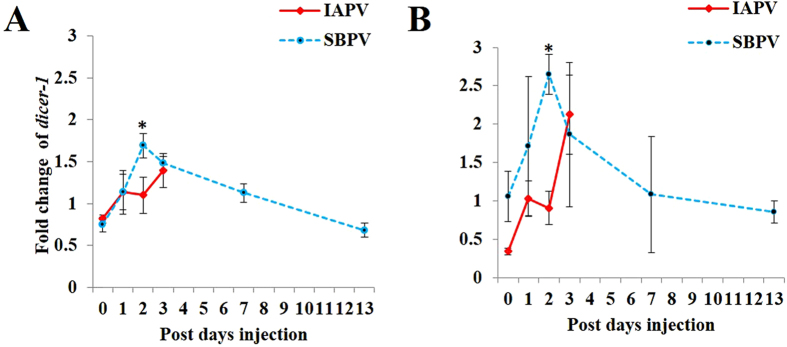
Fold changes of two core gene expressions in the miRNA pathway upon IAPV and SBPV infection. (**A**) Fold change of *dicer-1* expression upon viral infections; (**B**) Fold change of *ago-1* expression upon viral infections. The fold changes of gene expression were equal to the ratio of the relative expression of each gene in virus infected samples over the relative expression of this gene in control samples (PBS injected bees) (n = 4~5). The relative expression of each gene was calculated based on internal reference gene *PPIA*. The error bar represented the standard error of mean. The level of significance was calculated by t-test, and *p* < *0.0125* for the group of IAPV and *p *< *0.0083* for the group of SBPV was labeled with asterisks (*), respectively.

Aside from some non-canonical pathways of miRNA biogenesis, both Dicer-1 and Ago-1 are the core components of miRNA biogenesis and determine the pathway’s activity on gene regulation^6^. Here, we measured the expression of *B. terrestris dicer-1* and *ago-1* upon viral infections. In Niu *et al*.^2^, we showed that SBPV and IAPV replicated fast, but the two viruses differed largely in causing mortality in bees: SBPV as an avirulent virus and IAPV as an extremely virulent virus^2^. In the current study, we have separated PBS controls for the two viruses, thus we used independent t-test to separate means of the expressions of *dicer-1* and *ago-1*. For IAPV (four time points) and SBPV (six time points) the Bonferroni corrected *p* values were 0.0125 and 0.0083, respectively. Our results showed that only a noticeable increase of both *dicer-1* and *ago-1* was observed upon IAPV at 3 days post infection (dpi) (Fig. 1), while both *dicer-1* (t-test: t = 5.861, df = 8, *p* < *0.001*) and *ago-1* (t-test: t = 5.370, df = 4.578, *p* = *0.004*) were significantly upregulated at 2 dpi of SBPV (Fig. 1 and Table S1).

### Small RNA sequencing reveals differentially expressed miRNAs upon SBPV and IAPV infections

Above we presented indications that the expression of core components of the miRNA pathway could be altered upon viral infections in *B. terrestris*, especially after SBPV infection, which implied the potential involvement of the miRNA pathway in host-virus interaction. To explore this possibility further, we used small RNA sequencing to find out which miRNAs could be influenced. Currently, there are 217 miRNAs described for the honeybee *Apis mellifera* in miRBase, while in the two bumblebee species, *B. terrestris* and *B. impatiens*, 130 and 115 miRNAs have been identified, respectively^12^. Our results revealed that 17 and 12 host miRNAs were differentially expressed upon infection with SBPV and IAPV, respectively (Table 1, Table S2 and S3). From the 17 miRNAs differentially expressed in bumblebees infected with SBPV, 10 miRNAs were upregulated and 7 were downregulated. In bumblebees infected with IAPV, 12 miRNAs were differentially expressed, which included 7 upregulated and 5 downregulated miRNAs. Among all these differentially expressed miRNAs, 3 miRNAs (bte-miR-277, bte-bantam, and bte-miR-263a) were upregulated and 3 (bte-miR-3759, bte-miR-11, and bte-miR-24) were downregulated in both viral infections.

Table 1 summarizes the potential functions of the differentially abundant miRNAs based on the function of homologous miRNAs demonstrated in others insects. Two reappearing functions (apoptosis and energy homeostasis), known to be regulated by miRNAs, struck our attention as they have also been described to be important factors in virus-host interactions.

#### Apoptosis

Homologous miRNAs of bte-miR-263a, bte-bantam, and bte-miR-11, three commonly differentially regulated miRNAs during SBPV and IAPV infections, have been shown to be involved in apoptosis^13^,^14^,^15^. In addition, a homolog of bte-miR-263b, which is upregulated by SBPV infection but not by the infection of IAPV, has been linked with apoptosis^14^.

#### Energy homeostasis (insulin-associated)

A homolog of bte-miR-278, upregulated by IAPV infection, is involved in energy homeostasis resulting in the regulation of levels of insulin^16^ and regulation of the detoxification enzyme P450s^17^. Homologs of bte-miR-13a and -13b upregulated by IAPV and SBPV, respectively, regulate the expression of juvenile hormone (JH)^18^. JH plays an import role in regulation of insect growth and molting, which are also associated with ecdysone and insulin signaling pathways^19^. The homolog of another miRNA regulated by IAPV infection, bte-miR-305, was shown to regulate Notch and insulin pathways in the intestinal stem cells of the *Drosophila melanogaster* gut^20^.

### Validation of reference miRNAs upon different time points of viral infections by RT-qPCR

**Figure 2:**
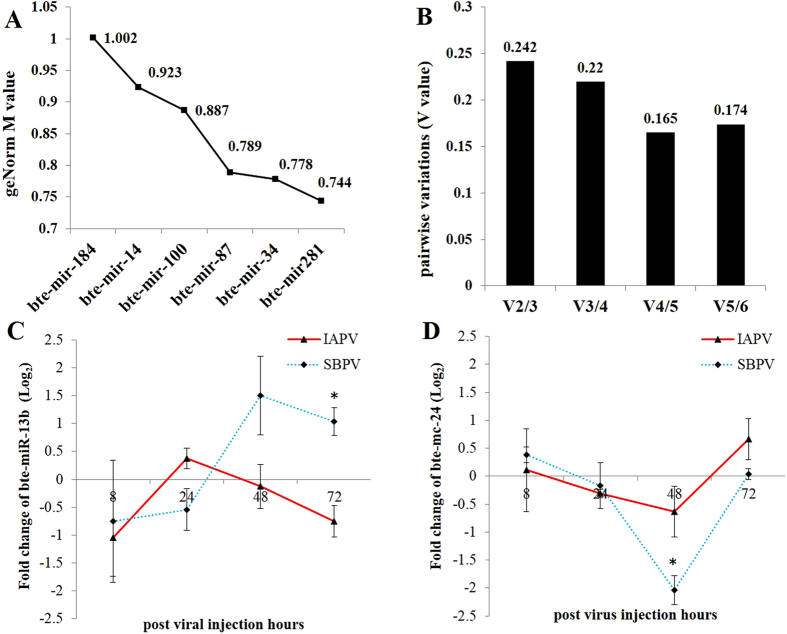
Validation of reference miRNAs upon different time points of IAPV and SBPV infections by RT-qPCR. (**A**) *M* value of the candidate reference miRNAs analyzed in samples of different time points of IAPV and SBPV infections by geNorm. (**B**) Pairwise variations (*V* value) for the combination of the candidate reference miRNAs by geNorm. (**C,D**) The fold changes of bte-miR-13b and bte-miR-24 expressions were calculated by the ratio of the relative expression of miRNA in virus infected samples over the relative expression of this miRNA in control samples (PBS injected bees) (n = 3). The relative expression of miRNA was calculated based on the combination of four optimal reference miRNAs: bte-miR-281 + bte-miR-34 + bte-miR-87 + bte-miR-100. The error bar represents the standard error of mean. The level of significance was calculated by t-test and *p *< *0.0125* was labeled with asterisks (*).

Until now, there have not been stable reference miRNAs known to normalize the data upon viral infections and non-infection in bumblebees. With the pre-selection criteria, based on primers efficiency and specificity (Table S4 and Figure S1), we eventually validated six miRNAs, including bte-miR-14, bte-miR-100, bte-miR-34, bte-miR-87, bte-miR-184 and bte-miR-281, in the same samples used to validate several key differentially expressed miRNAs indicated by small RNA sequencing. By geNorm, the results revealed that all these candidate reference miRNAs showed an *M* value of less than 1 except bte-miR-184, which showed an *M* value of 1.002 (Fig. 2A). Pairwise variations indicated the lowest *V* value of 0.165 (V4/5) upon the combination of the four most stable reference miRNAs (Fig. 2B). These results suggested the optimal references within these candidate reference miRNAs were the combination of four miRNAs, bte-miR-281 + bte-miR-34 + bte-miR-87 + bte-miR-100, for data analysis of miRNA expressions upon different injection time points of the two viruses.

To test the optimal reference miRNAs, we further evaluated the abundance of bte-miR-13b and bte-mc-24, the most potential miRNAs indicated by sequencing data to be involved in virus-host interaction, upon different time points after injection of the two viruses by RT-qPCR. The relative expressions of the miRNAs were normalized by four miRNAs selected above (miR-281 + bte-miR-34 + bte-miR-87 + bte-miR-100). At each time point, we separated PBS controls for two viruses, thus we used independent t-test to separate means but with Bonferroni correction as four comparisons for each virus were conducted, i.e. at four time points between virus injections and PBS injections. Therefore, the strict *p* value of 0.0125 was used to judge the statistical difference of miRNAs expressions. The fold change (log2 transformed) of miRNAs was calculated as the division of miRNAs expression in virus-injected samples by the very time points of PBS injected samples. The results showed that bte-miR-13b was upregulated at 48 and 72 h post-injection of SBPV, but only with significant difference at 72 h (*p* = *0.010*) (Fig. 2C). The significant downregulation of bte-mc-24 (*p* < *0.001*) was detected at 48 h post-injection of SBPV (Fig. 2D). These results were consistent with sequencing data and thereby these evaluated reference miRNAs could be used for further miRNA analysis upon virus infections.

### *In silico* target prediction of differentially expressed miRNAs shows a possible host-virus interaction network mediated by miRNAs

The current understanding is that one miRNA may target hundreds of genes, while a gene may be regulated by multiple miRNAs. Based on RNA22, we identified a total of 7465 genes possibly targeted by 130 miRNAs in *B. terrestris*. Among them, 7216 genes were possibly targeted by 23 differentially expressed miRNAs upon virus infection. Some predicted targets of these 23 miRNAs could be visualized in the GO and KEGG enrichments by ClueGO in two groups: 17 miRNAs differentially expressed by SBPV infection, and 12 miRNAs differentially expressed by IAPV infection. The analysis showed that there were 32 and 21 groups of GO/KEGG pathways in the network of SBPV (Figure S2) and IAPV (Figure S3). A number of 11 GO/KEGG pathways were common in the two datasets, which is consistent with the 6 common miRNAs influenced by SBPV and IAPV infections. Generally, these pathways can be associated with certain level of molecular activities in the view of virus-host interaction, such as DNA/RNA, protein, metabolism, cell activity, host disease, and host antiviral immunity (Table 2). These visualizations of miRNA-mRNA-GO/KEGG enrichments may show a possible host-virus interaction network regulated by virus-influenced miRNAs. Moreover, these potential targets reported in the homologous miRNAs, some of them also show an overlap with our predictions (Table 1). A future direction of research could be the role of apoptosis and insulin associated energy homeostasis in host-virus interaction based on miRNAs.

In addition, we also explored if host miRNAs could directly target viral genomic RNAs using RNAhybrid and RNA22. The results identified a total of 24 target sites on SBPV and IAPV genomes by host miRNAs (Table 3). The predicted target sites were located in the UTRs as well as ORFs (open reading frame). Of these 24 target sites, 16 were predicted to be targeted by differentially expressed miRNAs upon virus infections. We then applied a Chi square test to analyze whether these predictions, mainly linked with differential miRNAs, acted just by chance. We found a significant difference between the number of differentially expressed bumblebee miRNAs by each virus in targeting its own viral genomic RNAs and all bumblebee miRNAs in targeting viral genomic RNAs for both SBPV (χ^2^ = 14.816, *p* < *0.001*) and IAPV (χ^2^ = 13.422, *p* < *0.001*) analyses. This could suggest that the direct host miRNA-virus interplay might also play a role in bumblebee-SBPV/IAPV interaction. Intriguingly, a homolog of bte-miR-252a downregulated by SBPV, was shown to have a direct interaction with dengue virus (DENV) via targeting the DENV E protein transmembrane region^21^. Through RNAhybrid and RNA22, we also predicted that bte-miR-252a could possibly target the 3′UTR of SBPV (Table 3).

Further analyses are needed to elucidate these direct miRNAs-virus interaction, which may provide us with an alternative strategy to use miRNAs to control the viruses or in a combination with the strategy of virus specific small interfering RNA (siRNA) and dsRNA through the siRNA pathway^1^.

### Depletion of *dicer-1* by RNAi does not lead to an altered genome copy number of SBPV

**Figure 3:**
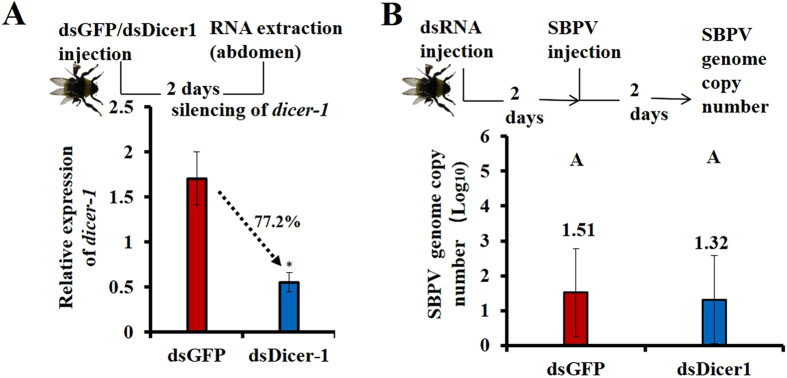
Silencing of *dicer-1* and its effect on the genome copy number of SBPV. (**A**) The injection of dsDicer-1 (n = 5) to target *dicer-1* led to 77.2% reduction of its transcript levels compared with injection of the negative control dsGFP (n = 5). (**B**) Pre-silencing of *dicer-1* led to no difference of genome copy number of SBPV (n = 12).

Although our results showed an altered expression of *dicer-1* and *ago-1* and differential abundance of miRNAs upon virus infections, the role of the miRNA pathway itself on the outcome of viral pathogenicity is not clear. Therefore, we used RNAi to silence *dicer-1* to detect its influence on the outcome of SBPV genome copy number (gcn), which was the avirulent infection and significantly influenced the expression of *dicer-*1, *ago-1* and miRNAs. Our result showed a ~77% depletion of *dicer-1* transcripts after two days of injection of dsRNA (Fig. 3A). However, silencing of *dicer-1* did not result in a significant difference in SBPV gcn (Fig. 3B). In our previous study, we also found that silencing of *dicer*-*2* had no effect on IAPV and SBPV viral gcn, although silencing efficiency in *dicer-2* depleted insects was reduced^2^. However, the answer to whether these results are associated with incomplete silencing of these key components from the RNAi pathway demands further study. In addition, it has been noted that various bumblebee tissues show different susceptibilities to viral infection^22^, but yet the virus-bumblebee interactions at the level of tissues rather than whole body or body parts awaits further studies. Recent studies showed that RNAi and JAK-STAT showed a cross-talk activity during viral infections^23^,^24^. In our previous study, we also detected the association between the RNAi and JAK/STAT pathways upon the infection of bee viruses in bumblebee^25^. Therefore, silencing of genes of the RNAi pathway could influence different (especially immune) pathways.

In conclusion, we found that an avirulent virus infection induced by SBPV and a virulent infection induced by IAPV in bumblebee *B. terrestris* could alter the miRNA pathway core genes’ expression and the abundance of miRNAs. It seems that based on the level of induction of *dicer-1* and *ago-1* and the number of differentially expressed miRNAs, the miRNA regulation was more disturbed or influenced after SBPV infection compared with IAPV infection. The potential target predictions and GO and KEGG enrichments were produced for initial visualization purposes and still lack any biological evidence, while it reflected the potential of bumblebee miRNAs that could be involved in various aspects of molecular activities in the view of virus-host interaction. In addition, some differentially expressed miRNAs upon virus infection may directly target the viral genome based on our prediction. Further, the selected reference miRNAs could be used for miRNA expression analysis through RT-qPCR. In summary, our study opens a new insight into bee-virus interactions meditated by the miRNA pathway. These initial data and observations could direct and facilitate our further studies to search a host miRNA-based strategy to tackle bee viruses.

## Methods

### Insects and virus inoculation

The colonies of *B. terrestris* were obtained from Biobest NV (Westerlo, Belgium). The bees used in this study were screened by RT-PCR (Table S5) to make sure that they were free of SBPV and IAPV. Newly emerged workers within 24 hours were genuinely collected from various colonies (within each experiment a maximum of 2 newly emerged workers were collected from a single colony, this was to ensure a random genetic background and to avoid overlap between treatment and genetic background) to keep the homogeneity of bees used in this study and kept in micro-colonies fed with pollen and sugar water *ad libitum* for further experiments. All the micro-colonies were maintained in an incubator (Panasonic, Japan) at 29–31 °C, 60–65% relative humidity, and continuous darkness^26^.

Virus inocula were produced as described previously^26^. The virus particles were counted by using transmission electron microscopy. To inoculate bees, we injected 20,000 and 20 particles of SBPV and IAPV into five to eight days old workers on the side of the soft white-like cuticle between the 1^st^ and 2^nd^ abdominal segments by a nanoinjector (Eppendorf, Germany) according to a previous study^2^. PBS (10 mM phosphate buffer pH 7.0/0.02% diethyl dithiocarbamate) injected bumblebees served as control, which was the same solution used to purify viral samples. Although both viruses replicate very fast, we define SBPV as an avirulent virus infection while IAPV represent a highly virulent infection as the injection of SBPV causes no mortality and IAPV causes extremely high mortality after 3 days and 100% mortality around 8 days^2^.

### RNA isolation, cDNA synthesis, and qPCR

A total of 1.5~2 ml RLT buffer was initially used to homogenize the individual bumblebee by mortar and the supernatant was centrifuged three times to remove debris. Thereafter, the protocol of the RNeasy mini kit (Qiagen, Germany) was followed. The TURBO DNA-free™ kit (Ambion, USA) was used to remove the possible genomic DNA contamination in RNA samples. The quantity and quality of RNA samples were checked by Nanodrop and electrophoresis on 1.5% agarose gel. An amount of 2 μg total RNA was used to synthesize the cDNA by SuperScript^®^ II Reverse Transcriptase (Invitrogen, USA) using oligo (dT) primers. To make sure there was no genomic DNA contamination, we checked cDNA samples by PCR with exon spanning primers for RPL23 (Table S5). The cDNA should produce an amplicon of 143 bp whereas the presence of genomic DNA will produce an extra amplicon of 452 bp. Next, qPCR was performed on a CFX96™ Real-Time PCR Detection with GoTaq^®^ qPCR master (Promega, USA). Each reaction was performed in duplicate. The amplification specificities of primers used in this study were checked by both electrophoresis of the RT-PCR products and analysis of the dissociation curve by qPCR. A 10-fold serial dilution of cDNA was applied to calculate the amplification efficiency (Table S5). In addition, the RT-PCR products were sequenced in order to confirm their primers’ amplification specificities.

### Core gene expression of the miRNA pathway

We collected RNA samples at 8 h, and 1, 2, 3, 7 and 13 dpi with SBPV and 8 h, and 1, 2, and 3 dpi with IAPV, to analyze gene expressions (n = 4~5). These time points were ascertained from previous study in consideration of the viral replication and viral caused mortality^2^. For each bumblebee individual, the whole body of each individual bee was used to extract RNA. PBS injected bees were also collected at all the different time points to serve as non-infected controls. Four to five bumblebee individuals were included in each time point for virus and PBS. The relative expression of key components, *dicer-1* and *ago-1*, of the miRNA pathway were normalized by internal control peptidylprolyl isomerase A (*PPIA*)^26^. The fold change of gene expression at each time point was given as the ratio of the relative gene expressions of the virus treated samples over the PBS controls collected at the same time point.

### Small RNA sequencing and target prediction of miRNAs

Small RNA sequencing was performed on RNA samples of SBPV and IAPV at 2 dpi, representing a fast replicating stage of IAPV and SBPV^2^. The PBS injected bees were included as control. For each bumblebee individual, the whole body of each individual bee was used to extract RNA. Four bumblebee individuals for each treatment and control were sequenced by the NXTGNT sequencing platform from the Ghent University. In detail, concentration and quality of the total extracted RNA was checked using the Quant-iT^TM^ RiboGreen^®^ RNA assay kit (Invitrogen, USA) and the RNA 6000 pico chip (Agilent Technologies, USA). Subsequently, 1 μg of total RNA was used to start the library preparation using the TailorMix miRNA Sample Preparation Kit v7 (SeqMatic, USA). Library preparation was carried out according to the manufacturer’s instructions. Then tRNA was added as carrier to minimize the loss of RNA via tube interaction. Libraries were quantified by qPCR, according to Illumina’s protocol ‘Sequencing Library qPCR Quantification protocol guide’ (version February 2011). A high sensitivity DNA chip (Agilent Technologies, USA) was used to check the libraries’ size distribution and quality. Single-end index 50 bp sequencing was performed on an Illumina MiSeq sequencer by loading 7 pM of each sample on the flowcell. A 10% PhiX spike-in was added as control. An average sequencing depth of 1.2 million reads in each sample was performed.

Ambiguous and low quality bases and adaptor sequences were trimmed from the sequencing reads using CLC Genomics Workbench 7.0.2. No ambiguous bases were allowed and a quality setting of 0.05 was applied. Reads smaller than 15 bp after trimming or reads containing more than 10% of bases with Phred quality score lower than 20 were filtered with CLC Genomics Workbench 7.0.2 and fastX-toolkit 0.0.13.1, respectively. The miRNA reads were counted by CLC Genomics Workbench 7.0.2 based on annotated bumblebee miRNA dataset^12^. Differential expression analysis between virus-infected and non-infected bees was performed in the R Bioconductor-package limma on quantile normalized data. The results were corrected by multiple testing using a Benjamini-Hochberg False Discovery Ratio at a cut-off value of 0.05. To select differentially expressed miRNAs, we used a strict criteria based on miRNA counts (greater than 100), fold change (larger than 20% difference), and adjusted *p* value (less than 0.01).

In this study, we screened the *B. terrestris* annotated mRNAs to identify potential miRNA binding sites using the core algorithm of improved version RNA22 v2^27^. This tool is publicly available from Computational Medicine Centre of Thomas Jefferson University (https://cm.jefferson.edu/rna22). RNA22 is a pattern based target prediction program which first searches for reverse complementary sites of patterns within given mRNA sequences and identifies the hot spots. In the next step, the algorithm searches for miRNAs that are likely to bind to these sites. We allowed maximum one mismatch in the seed region and minimum 12 nt matches in the entire binding site. We set the sensitivity and specificity thresholds at 63% and 61%, respectively.

To further view the potential targets of miRNAs with differential expressions upon viral infections, we built a network of enrichment of Gene Ontology (GO) terms or Kyoto Encyclopedia of Genes and Genomes (KEGG) pathways by ClueGO^28^. Briefly, ClueGO is a Cytoscape plug-in App to facilitate the visualization of functionally grouped terms in the form of networks and charts. We used a maximum of 25 predicted targets per miRNAs (folding energy cut-off: −20 Kcal/mol) as input. The GO term retrieved from Uniprot (ftp://ftp.ebi.ac.uk/pub/databases/GO/goa/UNIPROT/) and KEGG annotation (http://www.genome.jp/kegg/kegg2.html) of *B. terrestris* were input with ClueGO main id, Entrez gene ID. The default parameters of ClueGO 2.1.7 were used for network construction. In details, the GO term level was defined with minimum 3 and maximum 8; in each term cluster, it required a minimum of 3 genes with a minimum of 4.0% clustered genes over the total genes in the term. The enrichment of predicted target genes of miRNAs to GO term/KEGG pathway was tested based on the hypergeometric distribution. A further multiple testing was followed by Bonferroni step-down. Finally, ClueGO created a binary gene-term matrix with the selected terms and their associated genes. Based on this matrix, a term-term similarity matrix was calculated using chance corrected kappa statistics to determine the association strength between the terms. In the current analysis, we used a cut-off value 4.0 for kappa score, to represent the most significant (leading) terms in associated functional groups, which could be visualized in the network.

In addition, we also predicted the potential targets of *B. terrestris* miRNAs in SBPV and IAPV genomic RNA by RNA22^27^ and RNAhybrid^29^, which use different prediction assumptions and thus made our prediction more reliable. RNAhybrid is a tool for finding the normalized minimum free energy of hybridization for miRNA and their mRNA target genes. The small RNA sequence is hybridized to the best fitting part of the mRNA. We did not allow G:U pairing in the seed region (nucleotide 2–8) and forced miRNA-target duplexes to have a helix in this region. Maximum 5 nt were approved as unpaired nucleotides in either side of an internal loop. The outcomes of the two tools were merged, and miRNA binding sites that were predicted by both tools were considered highly confident.

### Validation and expression analysis of miRNAs by RT-qPCR

The same samples collected at 8, 24, 48, 72 h (n = 3) for both virus injections and PBS controls were used to further analyze the expressions of miRNAs. An amount of 2 μg total RNA was used to prepare cDNA by miScript^®^ II RT kit (QIAGEN, Germany). To quantify mature miRNAs in the study, the 5x miScript HiSpec buffer was used. The total volume of 20 μl reaction system was mixed by adding buffer, nucleic acids mix, reverse transcriptase mix, RNA template and RNase-free water, and then followed the protocol of the kit. For RT-qPCR, an volume of 20 μl reaction system was mixed by adding SYBR green PCR master mix, universal primer (provided by the kit), miRNA specific primer (Table S4), template cDNA prepared above and RNase free water, by following the protocol of miScript SYBR^®^ Green PCR kit (QIAGEN, Germany). Each reaction contained two technical replicates. To check the efficiency and specificity of miRNA primers, we evaluated the miRNAs’ amplification efficiencies by using a 10-fold serial dilution of mixture cDNA and the melting curves in all tested cDNA samples.

The validation of references as the same principle of mRNA expressions through RT-qPCR, was also suggested before the detection of miRNAs’ expressions^30^,^31^. To select good candidate reference miRNAs, we selected those with high counts (>100) and low expression variabilities indicated by sequencing data. Some miRNAs with significant changes upon viral infections revealed by the small RNA sequencing (section 2.4) were chosen to analyze their expressions in samples described above. The analysis of stable reference miRNAs was calculated by plug-in software geNorm in qBase^PLUS^ and the relative expressions of miRNAs were normalized by selected optimal reference miRNAs through qBase^PLUS^.

### Silencing *Dicer-1* and detection of its effect on viral genome copy number

The same protocol of RNAi to silence a specific gene was performed according to our previous study^2^. A fragment of *dicer-1* was amplified by PCR with target gene sequence specific primers plus T7 promoters (Table S5). This partial DNA template was purified by E.Z.N.A.^®^ Cycle-Pure Kit (Omega, USA), and was sequenced in order to confirm the identity of the amplification. Then, 1 μg of template was used to synthesize dsRNA according to the guideline of MEGAscript^®^ RNAi Kit (Invitrogen, USA). The concentration and quality of dsRNA were verified by Nanodrop and electrophoresis on 1.5% agarose gel. A total of 20 μg (20 μl) of dsRNA was injected into five to eight days old workers, and the same dose of dsGFP served as negative control. We used RNAi approach to investigate whether silencing of *dicer-1* influences the genome copy number (gcn) of SBPV. First we silenced *dicer-1* by injection of dsDicer1 (dsGFP serves as a control). After two days, the second injections were performed to inoculate bees with SBPV. Subsequently, in each sample, the abdomen of individual bumblebees was used for RNA extractions. To only measure the *dicer-1* silencing efficiency, five bumblebee individuals were used. For detecting the genome copy number of SBPV upon silencing of *dicer-1*, 12 bumblebee individuals were included. The whole experiment was repeated twice.

The measurement of SBPV gcn was based on a standard curve made from the Cq values (x) detected from a dilution of DNA templates of partial SBPV genome converting to corresponding gcn (y)^2^. In detail, a part of the SBPV genome (Table S5) was amplified and purified by E.Z.N.A.^®^ Cycle-Pure Kit (Omega, USA). The partial sequence was confirmed by Sanger sequencing (LGC genomics, Germany). The concentration of purified templates was measured by Quant-iT^TM^ PicoGreen^®^ dsDNA assay kit (Invitrogen, USA). The concentration was converted to gcn per μl by the online tool (URL: http://cels.uri.edu/gsc/cndna.html; Accessed date: 20/June/2014). A 10 fold serial dilution of templates was made to obtain a standard curve for each virus by qPCR. The equation is y = -0.2926x + 9.4426 (R^2^ = 0.9996). The normalized gcn of each sample was represented by the ratio of the gcn calculated based on the standard curve and the normalization factor from the internal reference gene *PPIA*^26^ with the framework of qBase^32^.

## Additional Information

**How to cite this article:** Niu, J. *et al*. Infections of virulent and avirulent viruses differentially influenced the expression of *dicer-1, ago-1,* and microRNAs in *Bombus terrestris.* *Sci. Rep.* **7**, 45620; doi: 10.1038/srep45620 (2017).

**Publisher's note:** Springer Nature remains neutral with regard to jurisdictional claims in published maps and institutional affiliations.

## Supplementary Material

Supplementary Data

## Figures and Tables

**Table 1:** Differentially expressed miRNAs upon virus infection.

miRNA name	fold change		Targets of homologous miRNA in insects and its relative prediction in *Bombus terrestris*	
	SBPV/PBS	IAPV/PBS	Homologous miRNA functions (target) in insects	Prediction of homologous targets or related targets in *Bombus terrestris*
bte-miR-13b	2.26		Juvenile hormone signaling pathway^18^	insulin-like growth factor 2 mRNA-binding protein 1-like (LOC100647990); ecdysone-induced protein 75B, isoforms C/D-like, (LOC100644185)
bte-miR-927a	1.75			
bte-miR-277	1.60	1.45	Branched-chain amino acid catabolism^33^ Neurodegeneration^34^	insulin receptor substrate 1-like (LOC100644779); insulin-degrading enzyme-like (LOC100644699)
bte-miR-263b	1.54		Apoptosis^14^	apoptosis-inducing factor 1, mitochondrial-like (LOC100651200); cell division cycle and apoptosis regulator protein 1-like (LOC100651179); apoptosis 2 inhibitor-like (LOC100647685); apoptosis regulator R1-like (LOC100649633); PRKC apoptosis WT1 regulator protein-like, transcript variant 1 (LOC100649375)
bte-bantam	1.49	1.28	Apoptosis^13^ Stem cell regulation^35^; Cell proliferation^36^ Tumor suppressor^37^	apoptosis 2 inhibitor-like (LOC100650673); SWI/SNF-related matrix-associated actin-dependent regulator of chromatin subfamily A containing DEAD/H box 1-like (LOC100645044); disks large 1 tumor suppressor protein-like (LOC100643982); fat-like cadherin-related tumor suppressor homolog (LOC100650960); cadherin-related tumor suppressor-like (LOC100651028)
bte-miR-9a	1.36		Wing development^38^ Transcription factor of senseless^39^,^40^;	neurogenic locus Notch protein-like (LOC100645932); strawberry notch-like (LOC100649094)
bte-miR-31a	1.32			
bte-miR-263a	1.26	1.42	Apoptosis^14^	apoptosis-inducing factor 1, mitochondrial-like (LOC100651200); cell division cycle and apoptosis regulator protein 1-like (LOC100651179); apoptosis-inducing factor 3-like, transcript variant 1 (LOC100649657)
bte-miR-10	1.26			
bte-miR-750	1.22			
bte-miR-305		1.30	Intestinal stem cells^20^	insulin-like growth factor-binding protein complex acid labile subunit-like (LOC100651887); insulin-degrading enzyme-like (LOC100644699); insulin-like peptide receptor-like (LOC100645413); insulin-like receptor-like (LOC100650952); insulin-like growth factor-binding protein complex acid labile subunit-like (LOC100643961)
bte-miR-13a		1.30	juvenile hormone signaling pathway^18^	insulin-like growth factor-binding protein complex acid labile subunit-like (LOC100647673); insulin-like receptor-like (LOC100651348); ecdysone-induced protein 75B, isoforms C/D-like (LOC100644185)
bte-miR-278		1.28	Energy homeostasis^16^ P450s 17	insulin-like peptide receptor-like (LOC100645413); insulin gene enhancer protein ISL-1-like (LOC100644486); cytochrome P450 18a1-like (LOC100642897); cytochrome P450 49a1-like (LOC100646342); cytochrome P450 4C1-like (LOC100651255); cytochrome P450 4g15-like (LOC100652170); cytochrome P450 6a13-like (LOC100646677); cytochrome P450 6a2-like (LOC100646434), (LOC100647785), (LOC100651291), (LOC100647041); cytochrome P450 6k1-like (LOC100642816), (LOC100642936), (LOC100643678), (LOC100647803), (LOC100648391), (LOC100648995), (LOC100650427); cytochrome P450 9e2-like (LOC100647566), (LOC100648545), (LOC100649871), (LOC100649988)
bte-miR-375		1.22		
bte-miR-252a	0.78		Dengue virus replication^21^	3′UTR of SBPV
bte-miR-316	0.72			
bte-miR-276	0.67		Olfactory^41^	putative odorant receptor 13a-like (LOC100647497); odorant receptor Or2-like (LOC100644343); putative odorant receptor 82a-like (LOC100646456); odorant receptor 47b-like (LOC100646577); putative odorant receptor 13a-like (LOC100646817); putative odorant receptor 63a-like (LOC100644217)
bte-miR-3718a	0.66			
bte-miR-3759	0.57	0.76		
bte-miR-11	0.56	0.61	Apoptosis^15^	DNA damage-binding protein 1-like (LOC100650523)
bte-mc-24	0.44	0.68		
bte-mc-753		0.75		
bte-miR-283		0.71		

**Table 2:** The most significant enriched GO/KEGG from the targets of differentially expressed miRNAs upon virus infection.

Diff. exp. miRNAs upon SBPV infection		Diff. exp. miRNAs upon IAPV infection	
GO/KEGG ID	name	GO/KEGG ID	name
**KEGG:00250**	**Alanine, aspartate and glutamate metabolism**	**KEGG:00250**	**Alanine, aspartate and glutamate metabolism**
**GO:0005524**	**ATP binding**	**GO:0005524**	**ATP binding**
**KEGG:04360**	**Axon guidance**	**KEGG:04360**	**Axon guidance**
**GO:0005975**	**carbohydrate metabolic process**	**GO:0005975**	**carbohydrate metabolic process**
**GO:0003924**	**GTPase activity**	**GO:0003924**	**GTPase activity**
**KEGG:05410**	**Hypertrophic cardiomyopathy (HCM)**	**KEGG:05410**	**Hypertrophic cardiomyopathy (HCM)**
**GO:0048519**	**negative regulation of biological process**	**GO:0048519**	**negative regulation of biological process**
**GO:0000166**	**nucleotide binding**	**GO:0000166**	**nucleotide binding**
**GO:0006082**	**organic acid metabolic process**	**GO:0006082**	**organic acid metabolic process**
**KEGG:03008**	**Ribosome biogenesis in eukaryotes**	**KEGG:03008**	**Ribosome biogenesis in eukaryotes**
**KEGG:05202**	**Transcriptional misregulation in cancer**	**KEGG:05202**	**Transcriptional misregulation in cancer**
GO:0016887	ATPase activity	GO:0006520	cellular amino acid metabolic process
GO:0016830	carbon-carbon lyase activity	GO:0005856	cytoskeleton
KEGG:04110	Cell cycle	GO:0003677	DNA binding
KEGG:04713	Circadian entrainment	GO:0006259	DNA metabolic process
GO:0006732	coenzyme metabolic process	GO:0004386	helicase activity
GO:0005794	Golgi apparatus	GO:0008237	metallopeptidase activity
KEGG:04390	Hippo signaling pathway	KEGG:04145	Phagosome
GO:0016788	hydrolase activity, acting on ester bonds	GO:0004672	protein kinase activity
KEGG:04630	Jak-STAT signaling pathway	GO:0038023	signaling receptor activity
GO:0000278	mitotic cell cycle	KEGG:04530	Tight junction
GO:0002009	morphogenesis of an epithelium		
GO:0001882	nucleoside binding		
GO:0031090	organelle membrane		
GO:0006508	proteolysis		
KEGG:00230	Purine metabolism		
KEGG:04015	Rap1 signaling pathway		
GO:0010646	regulation of cell communication		
GO:0010033	response to organic substance		
KEGG:04550	Signaling pathways regulating pluripotency of stem cells		
GO:0004888	transmembrane signaling receptor activity		
KEGG:05203	Viral carcinogenesis		

Notes: pathways with bold are the same GO/KEGG appeared in both SBPV and IAPV dataset. These GO/KEGG could be associated with certain biological functions in the view of host-virus interaction. Network of SBPV influenced miRNAs-targets-GO/KEGG: In DNA/RNA associated GO/KEGG, (GO:0005524), (GO:0003924), (GO:0000166), (GO:0016887), (GO:0001882), (KEGG:00230). In protein associated GO/KEGG, (KEGG:00250), (KEGG:03008), (GO:0005794), (GO:0006508). In metabolism associated GO/KEGG, (GO:0005975), (GO:0006082), (GO:0016830), (GO:0006732). In cell activity associated GO/KEGG, (KEGG:04110), (GO:0000278), (KEGG:04015), (GO:0010646), (GO:0010033), (KEGG:04550), (GO:0004888). In host disease associated GO/KEGG, (KEGG:05410), (KEGG:05202), (KEGG:05203). In host antiviral immunity associated GO/KEGG, (KEGG:04390), (KEGG:04630). Network of IAPV influenced miRNAs-targets-GO/KEGG: In DNA/RNA associated GO/KEGG, (GO:0005524), (GO:0003924), (GO:0000166), (GO:0003677), (GO:0006259), (GO:0004386). In protein associated GO/KEGG, (KEGG:00250), (KEGG:03008), (GO:0006520), (GO:0008237), (GO:0004672). In metabolism associated GO/KEGG, (GO:0005975), (GO:0006082). In cell activity associated GO/KEGG, (GO:0005856), (GO:0038023), (KEGG:04530). In host disease associated GO/KEGG, (KEGG:05410), (KEGG:05202). In host antiviral immunity Phagosome (KEGG:04145).

**Table 3:** Predicted targets of host miRNAs in the viral genomes.

Target	miRNA	MFE (Kcal/mol)	Binding Site Start Position	Target Gene Annotation
SBPV	bte-miR-276	−21.8	7261	Polyprotein_ORF
SBPV	bte-miR-276	−20.7	2285	Polyprotein_ORF
IAPV	bte-bantam	−25	4919	Polymerase Polyprotein_ORF
IAPV	bte-mc-24	−28.5	6219	Polymerase Polyprotein_ORF
IAPV	bte-mc-24	−28.1	6554	UTR
IAPV	bte-mc-24	−25	328	5′UTR
SBPV	bte-miR-252a	−29.6	9370	3′UTR
SBPV	bte-miR-9a	−25.1	3777	Polyprotein_ORF
IAPV	bte-miR-11	−21.8	7752	Structural Polyprotein_ORF
IAPV	bte-miR-283	−20.8	1145	Polymerase Polyprotein_ORF
IAPV	bte-mc-24	−22.5	9474	3′UTR
IAPV	bte-mc-753	−22.4	7878	Structural Polyprotein_ORF
SBPV	bte-miR-263b	−21.9	3705	Polyprotein_ORF
SBPV	bte-miR-276	−22.9	2500	Polyprotein_ORF
SBPV	bte-miR-3759	−23.3	7714	Polyprotein_ORF
SBPV	bte-mc-24	−20.1	9239	3′UTR
SBPV	bte-mc-753*	−26.3	7748	Polyprotein_ORF
IAPV	bte-miR-263b*	−24.2	8501	Structural Polyprotein_ORF
IAPV	bte-miR-9a*	−20.2	5318	Polymerase Polyprotein_ORF
IAPV	bte-miR-252a*	−22.2	3819	Polymerase Polyprotein_ORF
IAPV	bte-miR-252a*	−21.8	7091	Structural Polyprotein_ORF
IAPV	bte-miR-263b*	−20.1	6255	Polymerase Polyprotein_ORF
IAPV	bte-miR-750*	−21.9	1993	Polymerase Polyprotein_ORF
SBPV	bte-miR-305*	−22.7	785	Polyprotein_ORF

^*^Not differentially expressed due to that particular viral infection.
