# Supplementary material for: Infections of virulent and avirulent viruses differentially influenced the expression of dicer-1, ago-1, and microRNAs in Bombus terrestris
Source: Sci Rep. 2017 Apr 4;7:45620. doi: 10.1038/srep45620 (PMC5379481; doi:10.1038/srep45620)

Title

Infections of virulent and avirulent viruses differentially influenced the expression of *dicer-1*, *ago-1*, and microRNAs in *Bombus terrestris*

Jinzhi Niu[1,2], Ivan Meeus[2], Dieter IM De Coninck[3], Dieter Deforce[3], Kayvan Etebari[4], Sassan Asgari[4], Guy Smagghe[1,2,]*

[1]Key Laboratory of Entomology and Pest Control Engineering, College of Plant Protection, Southwest University, Chongqing 400716, China

[2]Department of Crop Protection, Faculty of Bioscience Engineering, Ghent University, Coupure Links 653, B-9000 Ghent, Belgium

[3]Laboratory for Pharmaceutical Biotechnology, Faculty of Pharmaceutical Sciences, Ghent University, Ottergemsesteenweg 460, B-9000 Ghent, Belgium.

[4]Australian Infectious Disease Research Centre, School of Biological Sciences, The University of Queensland, Brisbane, QLD 4072, Australia

*Corresponding author: guy.smagghe@ugent.be

Supplementary data

Table S1: p value of non-parametric test for all data sets by Mann-Whitney U

comparison factor	expression level				
	dicer-1	*ago-1*	bt-miR-13b	bt-mc-24	SBPV
IAPV vs. control at 8h	0.200	0.029	0.400	1.000	
IAPV vs. control at 24h	0.886	0.886	0.400	0.100	
IAPV vs. control at 48h	0.686	0.886	1.000	0.400	
IAPV vs. control at 72 h	0.229	0.114	0.400	0.400	
SBPV vs. control at 8h	0.200	0.886	1.000	0.200	
SBPV vs. control at 24h	0.686	0.343	0.200	0.700	
SBPV vs. control at 48h	0.004	0.004	0.200	0.050	
SBPV vs. control at 72 h	0.200	0.343	0.050	1.000	
SBPV vs. control at 7 days	0.886	1.000			
SBPV vs. control at 13 days	0.114	0.686			
dsDicer-1 vs. dsGFP	0.008				
dsDicer-1 vs. dsGFP (upon viral infection)					0.525

Table S2: data of miRNAs expression (IAPV infection samples vs. Control)

	Average counts							
miRNA	IAPV infected samples	PBS injected samples	AveExpr	log2(IAPV/PBS)	F	P.Value	adj.P.Val	fold
bte-miR-996	0.6563	0.0000	0.3281		1.4729	0.2970	0.3377	
bte-miR-980	0.2500	0.0000	0.1250		0.2317	0.7995	0.8281	
bte-miR-219	0.2188	0.0000	0.1094		0.1637	0.8524	0.8751	
bte-miR-307	1.5000	0.4063	0.9531	1.8845	4.2392	0.0665	0.0849	3.6923
bte-miR-7	3.0938	0.9375	2.0156	1.7225	4.1205	0.0700	0.0883	3.3000
bte-miR-124	11.5625	4.7188	8.1406	1.2930	2.7421	0.1371	0.1657	2.4503
bte-miR-3791	118.6250	57.1250	87.8750	1.0542	11.4036	0.0075	0.0122	2.0766
bte-let-7	127.6563	63.8125	95.7344	1.0004	3.4492	0.0955	0.1172	2.0005
bte-miR-932	0.5938	0.3125	0.4531	0.9260	0.8197	0.4815	0.5220	1.9000
bte-miR-137	6.8438	3.7500	5.2969	0.8679	17.3189	0.0025	0.0046	1.8250
bte-miR-6000a	26.7813	15.6250	21.2031	0.7774	28.3013	0.0006	0.0015	1.7140
bte-miR-2765	2.3438	1.5000	1.9219	0.6439	4.2349	0.0666	0.0849	1.5625
bte-mc-485	27.6875	18.5938	23.1406	0.5744	22.9077	0.0012	0.0023	1.4891
bte-mc-295	11.9063	8.0625	9.9844	0.5624	51.2601	0.0001	0.0003	1.4767
bte-miR-277	2105.4375	1448.6250	1777.0313	0.5394	162.6287	0.0000	0.0000	1.4534
bte-miR-190	28.1250	19.5000	23.8125	0.5284	42.9930	0.0002	0.0005	1.4423
bte-miR-263a	845.9375	597.4063	721.6719	0.5018	37.6277	0.0003	0.0007	1.4160
bte-miR-3719	0.9063	0.6563	0.7813	0.4657	3.5169	0.0924	0.1153	1.3810
bte-miR-993	12.0625	9.0313	10.5469	0.4175	14.7509	0.0039	0.0067	1.3356
bte-mc-476	1.2500	0.9375	1.0938	0.4150	3.4393	0.0959	0.1172	1.3333
bte-miR-305	392.4375	302.3125	347.3750	0.3764	37.7059	0.0003	0.0007	1.2981
bte-miR-13a	124.6875	96.2188	110.4531	0.3739	61.5787	0.0001	0.0002	1.2959

bte-miR-282	0.9688	0.7500	0.8594	0.3692	2.3032	0.1755	0.2057	1.2917
bte-bantam	160.3750	124.8438	142.6094	0.3613	309.1513	0.0000	0.0000	1.2846
bte-miR-278	204.1250	159.1250	181.6250	0.3593	364.3783	0.0000	0.0000	1.2828
bte-miR-375	381.5000	311.6563	346.5781	0.2917	51.4737	0.0001	0.0003	1.2241
bte-mc-762	37.2500	32.0000	34.6250	0.2192	39.3845	0.0002	0.0007	1.1641
bte-miR-2-2	90.1250	77.7188	83.9219	0.2137	94.3946	0.0000	0.0001	1.1596
bte-miR-2-3	89.9375	78.3438	84.1406	0.1991	69.7584	0.0000	0.0002	1.1480
bte-miR-10	4145.3750	3617.6250	3881.5000	0.1965	334.6406	0.0000	0.0000	1.1459
bte-miR-133	681.3125	597.2188	639.2656	0.1901	119.6428	0.0000	0.0001	1.1408
bte-miR-100	1473.9375	1299.9063	1386.9219	0.1813	280.3230	0.0000	0.0000	1.1339
bte-miR-318	22.5000	19.9375	21.2188	0.1744	9.1180	0.0131	0.0194	1.1285
bte-miR-281	671.6563	597.4063	634.5313	0.1690	137.0811	0.0000	0.0000	1.1243
bte-miR-927a	114.3750	102.5313	108.4531	0.1577	34.7117	0.0003	0.0009	1.1155
bte-miR-2-1	69.7188	62.5938	66.1563	0.1555	48.5910	0.0001	0.0004	1.1138
bte-miR-210	34.8125	31.4063	33.1094	0.1486	27.9729	0.0006	0.0015	1.1085
bte-miR-3730	3.9688	3.6250	3.7969	0.1307	8.8018	0.0142	0.0209	1.0948
bte-miR-279b	11.4063	10.4375	10.9219	0.1280	5.6336	0.0382	0.0522	1.0928
bte-miR-2796	363.3750	334.5625	348.9688	0.1192	30.3136	0.0005	0.0012	1.0861
bte-miR-3785	759.6250	701.4375	730.5313	0.1150	77.6846	0.0000	0.0001	1.0830
bte-miR-34	1218.7188	1140.5000	1179.6094	0.0957	101.5866	0.0000	0.0001	1.0686
bte-miR-13b	77.4375	72.6875	75.0625	0.0913	54.7551	0.0001	0.0003	1.0653
bte-miR-6001	1.5938	1.5000	1.5469	0.0875	7.5172	0.0205	0.0290	1.0625
bte-miR-252b	39.9063	37.8125	38.8594	0.0778	119.8155	0.0000	0.0001	1.0554
bte-miR-14	3033.4688	2891.7500	2962.6094	0.0690	509.3730	0.0000	0.0000	1.0490
bte-miR-306	171.0938	164.5938	167.8438	0.0559	191.1733	0.0000	0.0000	1.0395
bte-mc-65	86.5000	83.7813	85.1406	0.0461	21.3543	0.0014	0.0028	1.0325

bte-miR-6037	10.2188	10.1563	10.1875	0.0089	8.5665	0.0152	0.0220	1.0062
bte-miR-92b	291.6250	293.6875	292.6563	-0.0102	170.1823	0.0000	0.0000	0.9930
bte-miR-31a	136.4375	137.4688	136.9531	-0.0109	90.1142	0.0000	0.0001	0.9925
bte-miR-87	111.4375	112.7500	112.0938	-0.0169	104.7013	0.0000	0.0001	0.9884
bte-miR-1175	63.9063	64.8750	64.3906	-0.0217	27.4430	0.0007	0.0015	0.9851
bte-miR-3049	11.3750	11.5938	11.4844	-0.0275	10.4090	0.0095	0.0145	0.9811
bte-miR-3786	30.1563	30.7500	30.4531	-0.0281	35.7992	0.0003	0.0008	0.9807
bte-miR-275	836.9063	854.1250	845.5156	-0.0294	152.3295	0.0000	0.0000	0.9798
bte-miR-2788	10.4375	11.0938	10.7656	-0.0880	38.2609	0.0003	0.0007	0.9408
bte-miR-317	899.7500	962.6563	931.2031	-0.0975	88.9165	0.0000	0.0001	0.9347
bte-miR-316	146.0938	157.1563	151.6250	-0.1053	26.7425	0.0007	0.0016	0.9296
bte-miR-3718a	1816.1875	1960.8125	1888.5000	-0.1105	193.5426	0.0000	0.0000	0.9262
bte-miR-29b	3.6875	4.0000	3.8438	-0.1174	8.1374	0.0171	0.0245	0.9219
bte-miR-6065	0.3438	0.3750	0.3594	-0.1255	0.6417	0.5569	0.5981	0.9167
bte-miR-184	2806.4375	3062.4375	2934.4375	-0.1259	100.2293	0.0000	0.0001	0.9164
bte-miR-276	3033.4688	3311.0938	3172.2813	-0.1263	211.2123	0.0000	0.0000	0.9162
bte-miR-9a	143.9063	158.0313	150.9688	-0.1351	116.2396	0.0000	0.0001	0.9106
bte-miR-79	4.5625	5.0938	4.8281	-0.1589	13.4976	0.0049	0.0083	0.8957
bte-mc-314	10.0938	11.3125	10.7031	-0.1645	17.2568	0.0025	0.0046	0.8923
bte-mc-1030	1376.1875	1543.0938	1459.6406	-0.1651	12.6400	0.0058	0.0096	0.8918
bte-miR-3726	7.7813	8.7813	8.2813	-0.1744	4.7295	0.0541	0.0706	0.8861
bte-miR-71	22.6250	25.8438	24.2344	-0.1919	34.9020	0.0003	0.0009	0.8755
bte-miR-8	694.8750	810.1875	752.5313	-0.2215	445.6668	0.0000	0.0000	0.8577
bte-miR-125	242.8438	284.0000	263.4219	-0.2259	79.4006	0.0000	0.0001	0.8551
bte-mc-732	1.0938	1.2813	1.1875	-0.2283	5.1930	0.0451	0.0608	0.8537

bte-miR-12	91.0625	106.8125	98.9375	-0.2302	243.5173	0.0000	0.0000	0.8525
bte-miR-252a	127.8750	150.6875	139.2813	-0.2368	21.2427	0.0014	0.0028	0.8486
bte-miR-263b	434.9688	522.5938	478.7813	-0.2648	86.4417	0.0000	0.0001	0.8323
bte-miR-3477	215.4688	261.4063	238.4375	-0.2788	53.9310	0.0001	0.0003	0.8243
bte-miR-6053	0.8438	1.0313	0.9375	-0.2895	2.3180	0.1740	0.2057	0.8182
bte-miR-750	412.4688	510.4375	461.4531	-0.3074	72.4227	0.0000	0.0001	0.8081
bte-mc-64	8.5000	10.5313	9.5156	-0.3091	25.9391	0.0008	0.0017	0.8071
bte-miR-315	4.4063	5.7188	5.0625	-0.3761	23.5286	0.0011	0.0022	0.7705
bte-miR-3759	281.9375	368.5625	325.2500	-0.3865	25.5307	0.0008	0.0018	0.7650
bte-mc-753	111.7500	148.0938	129.9219	-0.4062	19.1376	0.0019	0.0037	0.7546
bte-miR-927b	2.0625	2.8125	2.4375	-0.4475	4.8698	0.0512	0.0674	0.7333
bte-miR-279a	25.1875	34.5938	29.8906	-0.4578	17.7286	0.0024	0.0044	0.7281
bte-miR-283	306.3750	429.7500	368.0625	-0.4882	90.2440	0.0000	0.0001	0.7129
bte-miR-1	54.2188	76.8125	65.5156	-0.5025	33.9036	0.0004	0.0009	0.7059
bte-miR-193	3.6875	5.4375	4.5625	-0.5603	15.1201	0.0036	0.0064	0.6782
bte-mc-24	140.9375	208.2500	174.5938	-0.5633	37.1216	0.0003	0.0007	0.6768
bte-mc-218	6.9688	10.4063	8.6875	-0.5785	16.1424	0.0030	0.0054	0.6697
bte-mc-712	13.5313	21.5000	17.5156	-0.6680	96.1909	0.0000	0.0001	0.6294
bte-miR-11	77.7500	127.5625	102.6563	-0.7143	68.1671	0.0000	0.0002	0.6095
bte-miR-2944	33.7813	55.8750	44.8281	-0.7260	11.1147	0.0080	0.0128	0.6046
bte-miR-981	1.0938	1.9063	1.5000	-0.8015	6.5325	0.0280	0.0386	0.5738
bte-miR-92c	783.9375	1388.4063	1086.1719	-0.8246	9.4271	0.0121	0.0182	0.5646
bte-miR-33	0.5625	1.0000	0.7813	-0.8301	2.2806	0.1779	0.2063	0.5625
bte-mc-1607	0.3125	0.5625	0.4375	-0.8480	1.0478	0.4034	0.4457	0.5556
bte-miR-965	1.6250	2.9688	2.2969	-0.8694	12.0789	0.0065	0.0106	0.5474

bte-miR-92a	102.5625	194.4688	148.5156	-0.9230	10.7648	0.0087	0.0135	0.5274
bte-miR-6040	13.6563	26.1250	19.8906	-0.9359	10.8447	0.0086	0.0134	0.5227
bte-mc-1154	16.8438	32.9063	24.8750	-0.9661	161.0136	0.0000	0.0000	0.5119
bte-miR-9c	5.1875	11.5313	8.3594	-1.1524	7.2878	0.0220	0.0307	0.4499
bte-miR-6039	0.5625	1.2813	0.9219	-1.1876	2.7089	0.1396	0.1670	0.4390
bte-miR-3770	0.1563	0.3750	0.2656	-1.2630	0.4617	0.6495	0.6849	0.4167
bte-miR-1000	0.2188	0.5313	0.3750	-1.2801	1.0983	0.3884	0.4332	0.4118
bte-miR-3715	0.5000	2.2813	1.3906	-2.1898	4.9902	0.0488	0.0650	0.2192
bte-miR-279c	0.9063	4.3125	2.6094	-2.2505	18.0663	0.0022	0.0043	0.2101
bte-miR-3727	0.1563	0.7500	0.4531	-2.2630	0.8779	0.4599	0.5032	0.2083
bte-miR-6052	4.2500	23.7188	13.9844	-2.4805	12.8539	0.0056	0.0093	0.1792
bte-miR-929	0.4063	2.4688	1.4375	-2.6033	1.5492	0.2819	0.3238	0.1646
bte-mc-37	0.1875	1.2500	0.7188	-2.7370	1.3456	0.3245	0.3655	0.1500
bte-miR-6012	0.0313	0.3750	0.2031	-3.5850	0.3734	0.7023	0.7339	0.0833
bte-miR-971	0.0000	0.5938	0.2969		0.6136	0.5701	0.6067	0.0000
bte-miR-6067	0.0000	0.1250	0.0625		0.0618	0.9406	0.9412	0.0000
bte-miR-928	0.0000	0.1250	0.0625		0.0612	0.9412	0.9412	0.0000
bte-miR-iab-4	0.0000	0.1250	0.0625		0.0612	0.9412	0.9412	0.0000

Table S3: data of miRNAs expression (SBPV infection samples vs. Control)

miRNA	Average counts SBPV infected samples	PBS injected samples	AveExpr	log2(SBPV/PBS)	F	P.Value	adj.P.Val	fold
bte-miR-996	0.8750	0.0000	0.4375		0.9130	0.4474	0.4943	
bte-miR-980	0.1250	0.0000	0.0625		0.0416	0.9595	0.9678	
bte-miR-219	0.0000	0.0000	0.0000		0.0000	1.0000	1.0000	
bte-miR-iab-4	1.3438	0.0313	0.6875	5.4263	1.4588	0.2999	0.3444	43.0000
bte-miR-928	0.8125	0.0313	0.4219	4.7004	0.6792	0.5398	0.5907	26.0000
bte-miR-307	4.1563	0.3125	2.2344	3.7334	6.8419	0.0253	0.0354	13.3000
bte-mc-1607	2.8750	0.4688	1.6719	2.6167	4.5152	0.0592	0.0754	6.1333
bte-miR-6067	0.2188	0.0625	0.1406	1.8074	0.1320	0.8786	0.8940	3.5000
bte-miR-315	16.6250	5.7813	11.2031	1.5239	122.4250	0.0000	0.0001	2.8757
bte-miR-7	1.9063	0.6875	1.2969	1.4713	3.4847	0.0939	0.1171	2.7727
bte-miR-282	1.6875	0.6875	1.1875	1.2955	4.5333	0.0587	0.0754	2.4545
bte-miR-13b	142.0313	62.8438	102.4375	1.1764	31.9000	0.0004	0.0013	2.2601
bte-miR-3730	8.0313	3.6563	5.8438	1.1353	18.7622	0.0020	0.0044	2.1966
bte-miR-6001	3.0625	1.5000	2.2813	1.0297	2.3085	0.1750	0.2093	2.0417
bte-miR-190	41.0625	20.2188	30.6406	1.0221	24.7571	0.0009	0.0023	2.0309
bte-miR-2765	2.9063	1.5000	2.2031	0.9542	5.4807	0.0405	0.0539	1.9375
bte-miR-927b	4.6875	2.5000	3.5938	0.9069	6.3923	0.0293	0.0400	1.8750
bte-miR-981	3.5625	1.9063	2.7344	0.9022	2.5311	0.1541	0.1862	1.8689
bte-miR-927a	166.5625	95.1250	130.8438	0.8082	15.6298	0.0033	0.0066	1.7510
bte-mc-753	245.5000	152.8750	199.1875	0.6834	12.1820	0.0064	0.0116	1.6059

bte-miR-277	2291.4688	1433.4375	1862.4531	0.6768	106.4015	0.0000	0.0001	1.5986
bte-miR-263b	705.6250	459.0938	582.3594	0.6201	29.7817	0.0005	0.0015	1.5370
bte-miR-279b	16.9063	11.2188	14.0625	0.5916	6.7665	0.0259	0.0358	1.5070
bte-bantam	175.2500	117.4688	146.3594	0.5771	129.4557	0.0000	0.0001	1.4919
bte-miR-2944	69.8750	47.0000	58.4375	0.5721	9.3318	0.0124	0.0194	1.4867
bte-miR-6000a	23.9375	16.3438	20.1406	0.5505	9.8457	0.0109	0.0179	1.4646
bte-miR-29b	5.6563	3.9063	4.7813	0.5341	10.3998	0.0095	0.0160	1.4480
bte-miR-6037	14.5000	10.4063	12.4531	0.4786	15.7244	0.0033	0.0066	1.3934
bte-miR-993	12.9063	9.3125	11.1094	0.4708	8.2325	0.0166	0.0248	1.3859
bte-miR-1175	79.1875	57.4688	68.3281	0.4625	21.1000	0.0015	0.0033	1.3779
bte-miR-33	1.3750	1.0000	1.1875	0.4594	5.1513	0.0458	0.0604	1.3750
bte-miR-9a	220.2188	161.9375	191.0781	0.4435	200.2784	0.0000	0.0001	1.3599
bte-mc-485	25.2813	18.8125	22.0469	0.4264	11.2272	0.0079	0.0140	1.3439
bte-miR-137	5.0313	3.7500	4.3906	0.4240	7.2445	0.0223	0.0319	1.3417
bte-miR-31a	179.2500	135.5000	157.3750	0.4037	22.7842	0.0012	0.0028	1.3229
bte-miR-6040	32.9375	25.6250	29.2813	0.3622	9.8083	0.0110	0.0179	1.2854
bte-miR-263a	683.7500	541.8750	612.8125	0.3355	15.0338	0.0037	0.0072	1.2618
bte-miR-10	5238.0000	4173.1875	4705.5938	0.3279	124.2695	0.0000	0.0001	1.2552
bte-miR-79	6.2813	5.0625	5.6719	0.3112	7.7959	0.0189	0.0277	1.2407
bte-miR-750	566.6563	464.0625	515.3594	0.2882	83.3454	0.0000	0.0003	1.2211
bte-miR-278	193.5938	161.6250	177.6094	0.2604	61.7734	0.0001	0.0004	1.1978
bte-mc-218	12.4375	10.6250	11.5313	0.2272	23.5534	0.0011	0.0026	1.1706
bte-mc-762	35.1250	30.4688	32.7969	0.2052	41.5781	0.0002	0.0008	1.1528
bte-mc-1030	1749.1250	1525.2813	1637.2031	0.1976	9.5977	0.0116	0.0186	1.1468
bte-miR-305	332.1250	289.7813	310.9531	0.1968	39.5032	0.0002	0.0008	1.1461

bte-miR-87	115.9375	103.9063	109.9219	0.1581	24.5009	0.0010	0.0024	1.1158
bte-miR-14	3180.3125	2855.5000	3017.9063	0.1554	281.4848	0.0000	0.0000	1.1137
bte-miR-3719	0.6563	0.5938	0.6250	0.1444	1.0955	0.3892	0.4383	1.1053
bte-miR-306	182.4375	165.6875	174.0625	0.1389	62.1539	0.0001	0.0004	1.1011
bte-miR-8	775.0625	727.6875	751.3750	0.0910	273.5957	0.0000	0.0000	1.0651
bte-miR-92b	305.5000	288.4688	296.9844	0.0828	57.7994	0.0001	0.0004	1.0590
bte-miR-2788	11.9688	11.3125	11.6406	0.0814	12.2168	0.0063	0.0116	1.0580
bte-miR-279a	33.5625	32.0000	32.7813	0.0688	30.8979	0.0005	0.0013	1.0488
bte-miR-13a	88.5313	85.8438	87.1875	0.0445	37.3626	0.0003	0.0009	1.0313
bte-miR-275	786.5625	769.0000	777.7813	0.0326	69.2319	0.0000	0.0003	1.0228
bte-miR-283	393.5313	388.7500	391.1406	0.0176	141.1461	0.0000	0.0001	1.0123
bte-miR-2-2	66.4375	65.8438	66.1406	0.0130	47.2105	0.0001	0.0006	1.0090
bte-miR-3477	260.4375	259.5313	259.9844	0.0050	42.7109	0.0002	0.0007	1.0035
bte-miR-932	0.2813	0.2813	0.2813	0.0000	0.3118	0.7426	0.7757	1.0000
bte-miR-34	1081.1563	1103.0313	1092.0938	-0.0289	46.4460	0.0001	0.0006	0.9802
bte-mc-732	1.1250	1.1563	1.1406	-0.0395	3.1454	0.1110	0.1370	0.9730
bte-miR-125	272.4063	280.4063	276.4063	-0.0418	61.3146	0.0001	0.0004	0.9715
bte-miR-252b	35.7188	37.0000	36.3594	-0.0508	44.7600	0.0002	0.0007	0.9654
bte-miR-1	65.3438	67.7500	66.5469	-0.0522	17.4173	0.0025	0.0052	0.9645
bte-miR-375	295.1563	306.0938	300.6250	-0.0525	36.1381	0.0003	0.0010	0.9643
bte-miR-3049	11.7188	12.1875	11.9531	-0.0566	5.0310	0.0480	0.0626	0.9615
bte-miR-3791	50.2813	52.3750	51.3281	-0.0589	7.2420	0.0223	0.0319	0.9600
bte-miR-281	514.2500	541.8750	528.0625	-0.0755	149.9479	0.0000	0.0001	0.9490
bte-miR-12	89.5625	97.4375	93.5000	-0.1216	59.1273	0.0001	0.0004	0.9192
bte-miR-133	493.0313	539.5000	516.2656	-0.1299	58.3084	0.0001	0.0004	0.9139
bte-miR-	585.7188	641.9063	613.8125	-0.1322	43.5025	0.0002	0.0007	0.9125

3785								
bte-mc-64	9.6563	10.5938	10.1250	-0.1337	12.8222	0.0056	0.0108	0.9115
bte-miR-318	16.7813	18.8125	17.7969	-0.1648	5.5525	0.0394	0.0531	0.8920
bte-miR-92c	1182.0000	1347.1875	1264.5938	-0.1887	8.7049	0.0146	0.0223	0.8774
bte-miR-2-1	46.5938	53.4375	50.0156	-0.1977	31.2393	0.0005	0.0013	0.8719
bte-miR-2796	269.1875	310.1250	289.6563	-0.2042	38.3590	0.0003	0.0009	0.8680
bte-miR-2-3	58.5625	67.8438	63.2031	-0.2122	38.3221	0.0003	0.0009	0.8632
bte-miR-3786	25.7500	29.9375	27.8438	-0.2174	33.3117	0.0004	0.0012	0.8601
bte-miR-100	1101.6875	1290.4688	1196.0781	-0.2282	73.7313	0.0000	0.0003	0.8537
bte-miR-965	2.3438	2.7500	2.5469	-0.2306	8.2373	0.0166	0.0248	0.8523
bte-miR-184	2855.5000	3354.1250	3104.8125	-0.2322	43.0768	0.0002	0.0007	0.8513
bte-let-7	46.0938	55.6563	50.8750	-0.2720	70.8976	0.0000	0.0003	0.8282
bte-miR-210	24.8438	30.1563	27.5000	-0.2796	12.5013	0.0060	0.0113	0.8238
bte-miR-317	732.0313	892.1250	812.0781	-0.2853	56.6147	0.0001	0.0004	0.8205
bte-mc-65	62.4688	77.3125	69.8906	-0.3076	15.7752	0.0032	0.0066	0.8080
bte-miR-3726	7.6875	9.5313	8.6094	-0.3102	3.0359	0.1174	0.1434	0.8066
bte-mc1154	26.5938	33.2500	29.9219	-0.3223	132.7131	0.0000	0.0001	0.7998
bte-miR-252a	110.0000	141.3750	125.6875	-0.3620	18.3134	0.0022	0.0046	0.7781
bte-miR-316	112.5313	157.0938	134.8125	-0.4813	26.8605	0.0007	0.0019	0.7163
bte-miR-3770	0.2188	0.3125	0.2656	-0.5146	0.3024	0.7490	0.7757	0.7000
bte-miR-71	17.7500	26.4375	22.0938	-0.5748	10.5643	0.0091	0.0156	0.6714
bte-miR-276	2391.3750	3574.6563	2983.0156	-0.5800	54.6232	0.0001	0.0004	0.6690
bte-miR-3718a	1291.7188	1968.5000	1630.1094	-0.6078	79.1146	0.0000	0.0003	0.6562
bte-miR-9c	7.3750	11.8750	9.6250	-0.6872	6.9618	0.0243	0.0344	0.6211
bte-mc-314	7.1875	11.6250	9.4063	-0.6937	9.1731	0.0129	0.0199	0.6183
bte-mc-712	13.3125	22.9688	18.1406	-0.7869	50.6092	0.0001	0.0005	0.5796
bte-miR-	13.8125	23.9688	18.8906	-0.7952	10.8327	0.0086	0.0149	0.5763

6052								
bte-miR-3759	194.2813	341.4375	267.8594	-0.8135	34.5980	0.0003	0.0011	0.5690
bte-miR-279c	2.3750	4.1875	3.2813	-0.8182	22.0791	0.0013	0.0030	0.5672
bte-miR-11	69.1875	124.4688	96.8281	-0.8472	39.2298	0.0002	0.0008	0.5559
bte-miR-1000	0.1875	0.3438	0.2656	-0.8745	0.3750	0.7013	0.7463	0.5455
bte-miR-92a	105.4375	198.4375	151.9375	-0.9123	10.8555	0.0085	0.0149	0.5313
bte-miR-124	2.3125	4.5313	3.4219	-0.9705	20.3761	0.0016	0.0036	0.5103
bte-mc-295	4.0313	8.0938	6.0625	-1.0056	12.4397	0.0061	0.0113	0.4981
bte-mc-24	90.7813	205.9375	148.3594	-1.1817	29.4330	0.0006	0.0015	0.4408
bte-mc-476	0.3438	0.8438	0.5938	-1.2955	1.5068	0.2902	0.3366	0.4074
bte-miR-3715	0.7188	2.1563	1.4375	-1.5850	3.8559	0.0789	0.0995	0.3333
bte-miR-6053	0.2813	0.9688	0.6250	-1.7843	1.5263	0.2864	0.3355	0.2903
bte-miR-6039	0.3125	1.2188	0.7656	-1.9635	1.9677	0.2149	0.2544	0.2564
bte-mc-37	0.2188	1.1563	0.6875	-2.4021	1.0662	0.3979	0.4438	0.1892
bte-miR-193	0.5938	5.5625	3.0781	-3.2278	9.3626	0.0123	0.0194	0.1067
bte-miR-3727	0.0313	0.6875	0.3594	-4.4594	0.5923	0.5805	0.6293	0.0455
bte-miR-929	0.0000	2.3438	1.1719		1.2814	0.3397	0.3864	0.0000
bte-miR-971	0.0000	0.5938	0.2969		0.5128	0.6212	0.6673	0.0000
bte-miR-6012	0.0000	0.4063	0.2031		0.3212	0.7362	0.7757	0.0000
bte-miR-6065	0.0000	0.3125	0.1563		0.2497	0.7862	0.8071	0.0000

Table S4: Specific primers to detect miRNA expression by RT-qPCR.

microRNA	specific primer	amplification efficiency
bte-mir-13b	TATCACAGCCATTTTTGACGATT	1.992
bte-mc-24	TGTGGGGCGGCGTCCGGGTCACT	2.092
bte-mir-184	TGGACGGAGAACTGATAAGG	2.076
bte-mir-14	GGGGGTGAGAAACTGGCTTGGCT	2.076
bte-mir-100	AACCCGTAGATCCGAACTTGTG	2.011
bte-mir-87	GTGAGCAAAGTTTCAGGTGTGT	2.009
bte-mir-34	TGGCAGTGTTGTTAGCTGGTTGTG	2.017
bte-mir-281	AAGAGAGCTATCCATCGACAGT	2.057

Table S5: Other primers used in this study

Gene	Forward primer	Reverse Primer	Amplification efficiency (AE)	NCBI accession number	reference
PPIA （qPCR）	TCGTAATGGAGTTGAGGAGTGA	CTTGGCACATGAAGTTTGGAAT	1.988	XM_003402218.2	(Niu et al., 2014)
RPL23 （qPCR）	GGGAAAACCTGAACTTAGGAAAA	ACCCTTTCATTTCTCCCTTGTTA	1.985	XM_003400707.2	(Niu et al., 2014)
IAPV (amply partial genome)	CACCAATCACGGACCTCACA	ACAGTGTTAGCTGCAGGACA		EU436443.1	
IAPV (qPCR)	CCATGCCTGGCGATTCAC	CTGAATAATACTGTGCGTATC		EU436443.1	(de Miranda et al., 2010)
SBPV (amply partial genome)	CCAGGATCGCATCCCCTTAG	TGTTGTCTCCCACCTCATGC		GU938761.1	
SBPV (qPCR)	TCCAAGAGCAAGTATGCGGG	AGCATCAAAGCTAATTGCGGA		GU938761.1	
dicer-1 （qPCR）	AGAGGAACAAATTTCAAATGACGA	CTGATTCACACGCAGGGGAT	1.931	XM_003401907.2	
dicer-1 （dsRNA synthesis）	TAATACGACTCACTATAGGGAGATTGGTCAAGCGACATGGCAA	TAATACGACTCACTATAGGGAGAAATGAGCTTGTCTATGCTTCGT		XM_003401907.2	
ago-1（qPCR）	AGTAGTGGGGTCTTTCTCGC	TGCTCGCACTTATTTTACAATGACA	2.069	XM_012315499.1	

de Miranda, J.R., Cordoni, G., Budge, G., 2010. The Acute bee paralysis virus–Kashmir bee virus–Israeli acute paralysis virus complex. J. Invertebr. Pathol. 103, Supplement, S30-S47.

Niu, J., Cappelle, K., de Miranda, J.R., Smagghe, G., Meeus, I., 2014. Analysis of reference gene stability after Israeli acute paralysis virus infection in bumblebees *Bombus terrestris*. J. Invertebr. Pathol. 115, 76-79.

Figure S1: Melt curves of miRNA primers by RT-qPCR performed in all samples.

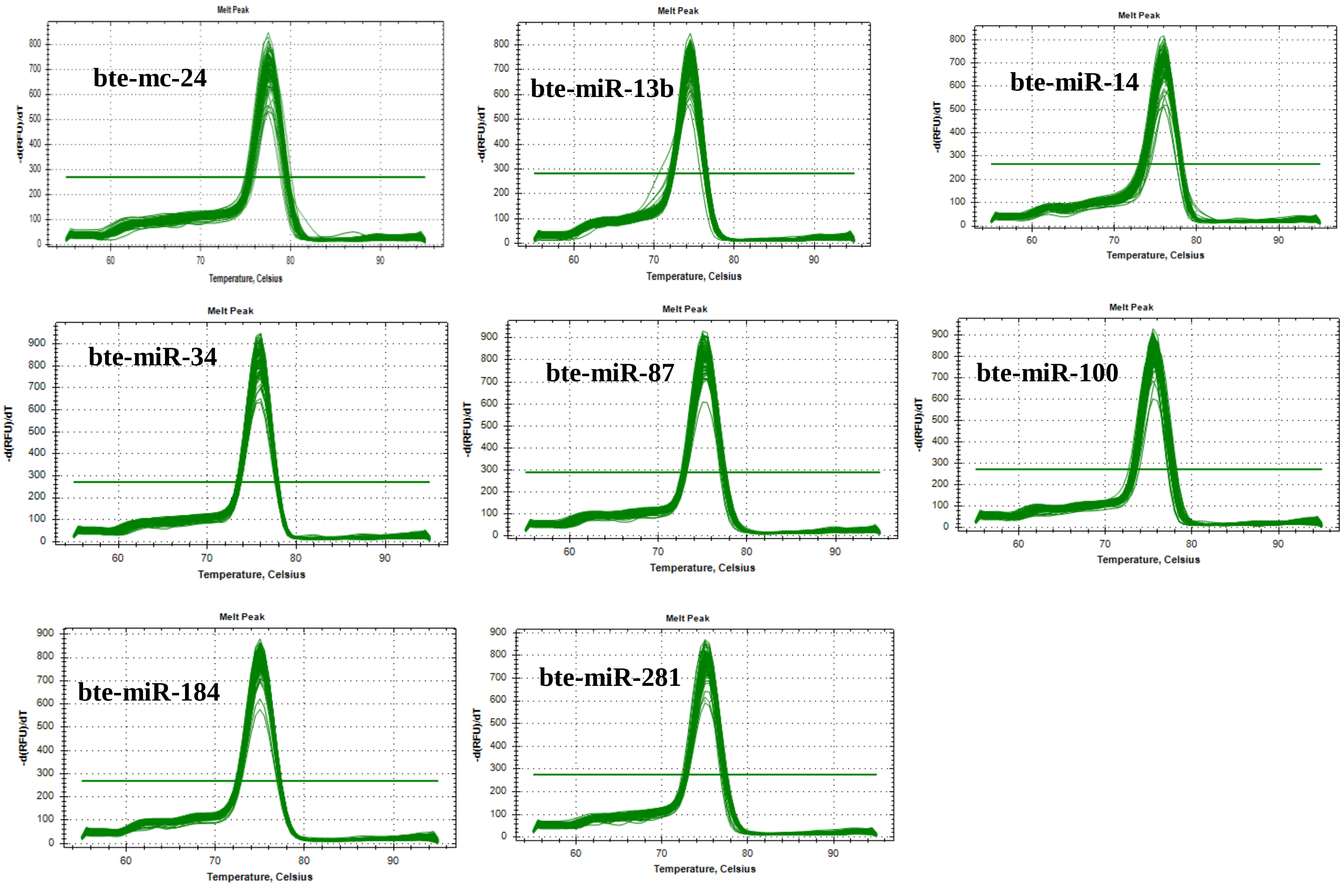

Figure S2: The enrichment of Gene Ontology (GO) terms and Kyoto Encyclopedia of Genes and Genomes (KEGG) pathways of predicted targets of differentially expressed miRNAs by SBPV infection. For each miRNA, a maximum of 25 predicted targets (folding energy cut-off: -20 Kcal/mol) was used as input to ClueGO. The GO and KEGG presented in the figure were based on Kappa score higher than 0.4.

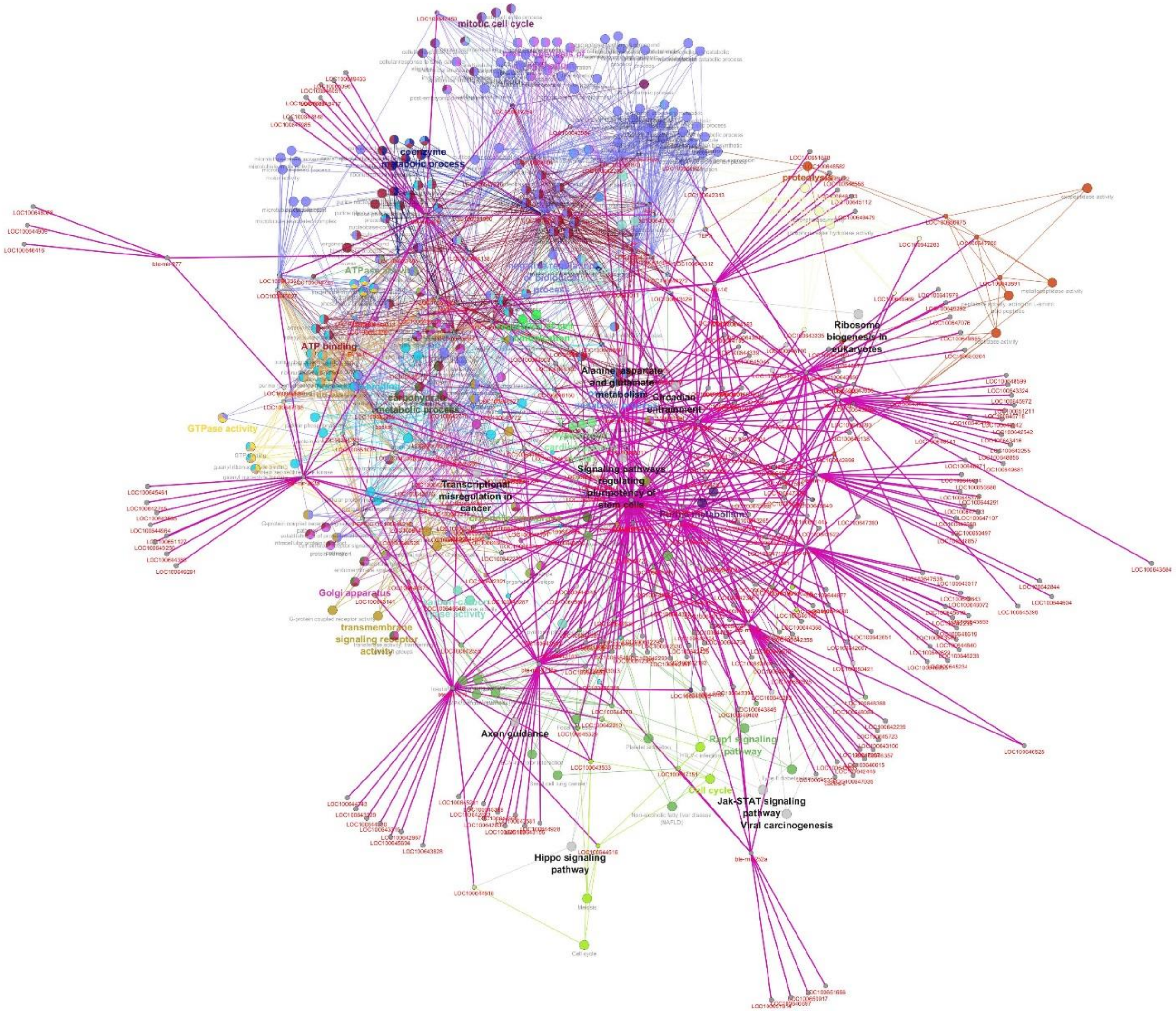

Figure S3: The enrichment of Gene Ontology (GO) terms and Kyoto Encyclopedia of Genes and Genomes (KEGG) pathways of predicted targets of differentially expressed miRNAs by IAPV infection. For each miRNA, a maximum of 25 predicted targets (folding energy cut-off: -20 Kcal/mol) was used as input to ClueGO. The GO and KEGG presented in the figure were based on Kappa score higher than 0.4.

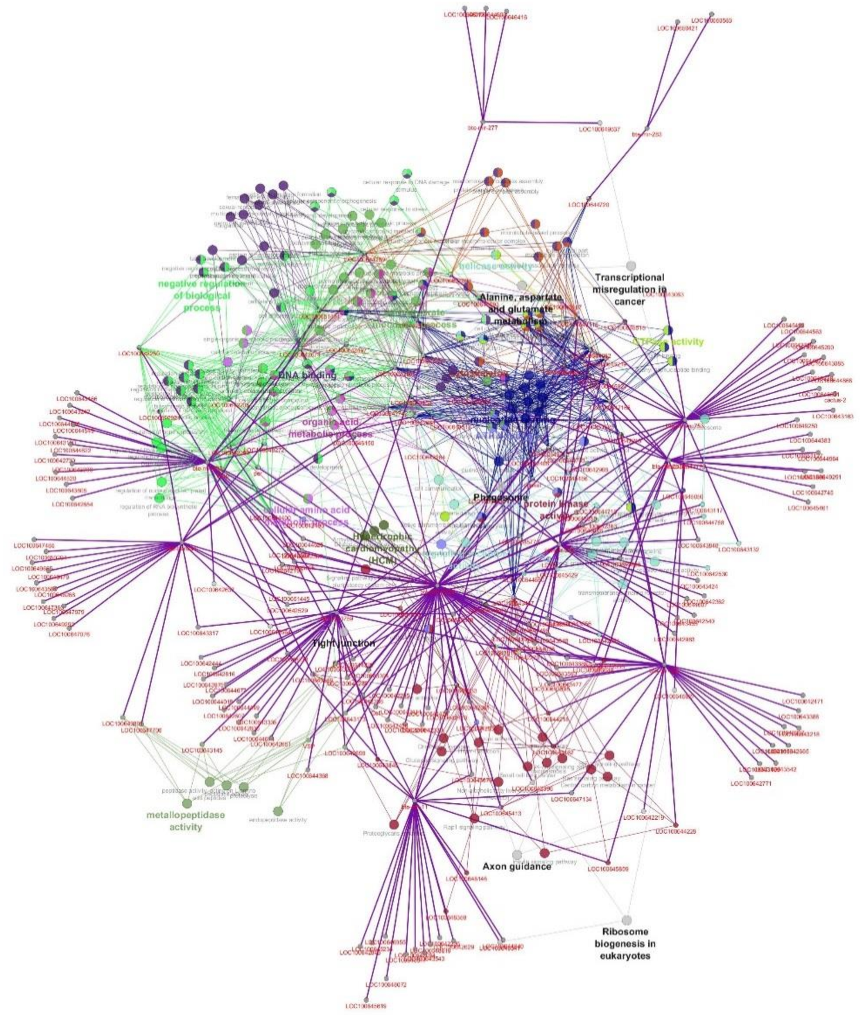

Supplement: Supplementary Data [file srep45620-s1.pdf]
